# Managing psychological distress in patients with colorectal cancer: A narrative review of nursing interventions

**DOI:** 10.1097/MD.0000000000044148

**Published:** 2025-09-05

**Authors:** Ping Ding, Qingbo Zhou, Jiaying Hu

**Affiliations:** a Central Sterile Supply Department, Shaoxing Yuecheng People’s Hospital, Shaoxing, China; b Resident Physician Standardized Training Base, Shaoxing People’s Hospital, Shaoxing, China; c Comprehensive Ward, Shaoxing Yuecheng People’s Hospital, Shaoxing, China.

**Keywords:** colorectal cancer, mental health support, nursing interventions, psychological distress, quality of life

## Abstract

This narrative review synthesizes evidence on effective nursing interventions aimed at mitigating the primary psychological challenges, particularly anxiety and depression, faced by patients with colorectal cancer. A literature search of key databases, including PubMed, Web of Science, and Google Scholar, was conducted for articles published between 2015 and 2024. The literature confirms that psychological distress is highly prevalent in colorectal cancer patients, with rates often ranging from 20% to 40%. Effective nursing interventions with demonstrated effectiveness include routine distress screening, patient education, psychological counseling, cognitive behavioral therapy, and promoting physical exercise, all of which are shown to significantly improve quality of life. Given that nurses play a central role in identifying and managing this distress, integrating structured screening and evidence-based supportive interventions into routine cancer care is critical. Future research should focus on developing personalized care protocols for high-risk subgroups, such as patients with ostomies or advanced disease.

## 1. Introduction

Colorectal cancer (CRC) ranks as one of the most significant oncological challenges worldwide, imposing a substantial burden not only on physical health but also on the psychosocial well-being of patients.^[1]^ Beyond the physiological impacts of the disease and its treatments, a large proportion of individuals with CRC experience significant psychological distress, most commonly manifesting as anxiety and depression.^[2]^ Evidence indicates that the prevalence of these conditions in CRC patients can range from 20% to 40%, varying by disease stage, treatment complexity, and individual patient factors.^[3,4]^

This psychological distress is not a secondary concern; it is intrinsically linked to crucial clinical outcomes. A growing body of literature demonstrates that untreated anxiety and depression in CRC patients are associated with a poorer health-related quality of life, reduced adherence to demanding treatment regimens, and potentially compromised long-term survival.^[5]^ Factors such as the stage of the cancer, the type of treatment received, and the patient’s inherent coping mechanisms can further exacerbate these mental health challenges, highlighting the need for targeted support.^[6]^

Within the oncology care continuum, nurses are uniquely positioned on the front lines to identify, monitor, and manage these psychological issues.^[7,8]^ Given their frequent and sustained contact with patients and their families, nurses are pivotal in providing holistic care that addresses both the physical and emotional dimensions of the cancer experience.^[9]^ However, for this role to be effective, nursing practice must be guided by evidence-based interventions tailored to the specific needs of the CRC population.

Therefore, this narrative review aims to synthesize the current literature on the primary psychological challenges affecting patients with CRC. Specifically, it will identify the most common forms of distress and evaluate the evidence for nursing interventions designed to manage these conditions, with the ultimate goal of improving patient quality of life and overall well-being.

## 2. Methodology

### 2.1. Study design

This manuscript is a narrative review, designed to provide a comprehensive overview and synthesis of the current state of knowledge regarding psychological distress in patients with CRC and the role of psychosocial interventions and their implications for nursing. It is not a systematic review. Therefore, a PRISMA flowchart and formal quality appraisal of individual studies were not performed. The primary goal is to summarize and critically discuss the literature from a clinical and nursing perspective, identifying key themes, evidence gaps, and directions for future practice.

### 2.2. Literature search and selection process

A literature search was conducted using the electronic databases PubMed, Web of Science, and Google Scholar to identify relevant publications. The search was focused on articles published between January 2015 and December 2024 to ensure the review reflects the most current research landscape, a key concern raised by reviewers.

The search strategy employed a combination of the following keywords: (“colorectal cancer” OR “CRC”) AND (“psychological distress” OR “anxiety” OR “depression” OR “mental health” OR “psychosocial support”) AND (“nursing intervention” OR “psychological intervention” OR “supportive care”).

The selection of literature was guided by the following criteria:

- Inclusion: Studies were included if they were published in English and focused on adult patients with CRC. The content needed to address the psychological aspects of the disease or evaluate nursing-led or psychologically-informed interventions. Preference was given to high-level evidence such as systematic reviews, meta-analyses, and randomized controlled trials (RCTs), as well as significant qualitative studies that provided deep insights into the patient experience.
- Exclusion: Studies were excluded if they focused on pediatric populations, cancers other than CRC, or if they were non-peer-reviewed articles, case reports, or editorials. Articles for which the full text was not accessible were also excluded.

The final selection of articles was based on their direct relevance to the central themes of this review, as determined by the authors.

As this is a narrative review synthesizing existing published literature and does not involve human participants, data collection, or any form of intervention, ethical approval from an institutional review board was not required. Similarly, informed consent was not applicable.

### 2.3. Manuscript preparation

To improve the clarity and grammatical quality of the manuscript, especially in response to reviewer feedback on writing issues, we utilized an AI language model (Grok, powered by xAI) for assistance with language polishing and phrasing suggestions during the revision process. All scientific content, interpretations, and decisions remain the sole responsibility of the authors, with no AI involvement in generating original ideas, data, or conclusions.

## 3. Synthesis and discussion of findings

The literature selection process identified 5 pivotal studies published between 2021 and 2024, which form the evidence base for this review. This final selection comprises high-level evidence, including 3 RCTs and 2 systematic reviews with meta-analyses, originating from China, Singapore, the Netherlands, and a European multicenter context. The detailed characteristics of these studies are summarized in Table 1. Synthesizing these studies reveals 3 core themes, which will be presented and discussed sequentially: The Confirmed Efficacy of Structured Psychological Therapies; The Role and Nuances of Technology-Mediated Interventions; and The Critical Distinction Between General Psychoeducation and Targeted Therapy.

**Table 1:** Summary of key studies on psychological distress and interventions in colorectal cancer.

Author & year	Country	Study design	Population	Intervention/focus	Key findings
Zhang X et al (2023)^[10]^	China	Systematic review & meta-analysis	12 RCTs involving 1231 CRC patients	Impact of psychoeducational interventions on patients	Psychoeducation significantly improved quality of life (QoL) in CRC patients, but the effects on anxiety and depression were not significant
Xia S (2023)^[11]^	(Not specified, but study conducted in a Chinese context)	Randomized controlled trial (RCT)	90 CRC patients who underwent tumor resection	Cognitive behavioral stress management (CBSM)	At 3 months post-surgery, the CBSM intervention group had significantly lower anxiety and depression scores and significantly higher quality of life compared to the control group
Wan SW et al (2022)^[12]^	Singapore	Systematic review & meta-analysis	10 Studies involving 1261 CRC patients	Web-based psychosocial interventions	Web-based interventions are effective in reducing depression, cancer-specific distress, and improving quality of life
Custers JAE et al (2024)^[13]^	The Netherlands	Randomized controlled trial (RCT)	165 Distressed CRC survivors	A blended (online + face-to-face) cognitive behavioral therapy (CBT)	Compared to usual care, blended CBT significantly reduced psychological distress (HADS total score), with effects sustained at 6 months
Maguire R et al (2021)^[14]^	European multicenter	Randomized controlled trial (RCT)	827 Cancer patients (including CRC) receiving chemotherapy	Real time remote symptom monitoring via the ASyMS system	The intervention group showed significant improvement in self-efficacy, but no significant differences in quality of life, anxiety, or depression compared to the control group

ASyMS = advanced symptom management system, CBSM = cognitive behavioral stress management, CBT = cognitive behavioral therapy, CRC = colorectal cancer, HADS = Hospital Anxiety and Depression Scale, QoL = quality of life, RCT = randomized controlled trial.

### 3.1. The confirmed efficacy of structured psychological therapies

The evidence provides strong confirmation that specific, structured psychological therapies are effective in reducing distress among CRC patients. An RCT in the Netherlands by Custers JAE et al (2024) demonstrated that a blended cognitive behavioral therapy (CBT) intervention led to a statistically significant reduction in psychological distress, with effects sustained at 6 months^[13]^ This is powerfully supported by an RCT from China (Xia S., 2023), which found that a cognitive behavioral stress management (CBSM) program resulted in patients having “significantly lower anxiety and depression scores and significantly higher quality of life” compared to a control group.^[11]^

The clear implication of these findings is that for nursing practice to be effective, it must move beyond providing only general supportive listening. These high-quality RCTs provide robust, current evidence that to achieve clinically significant improvements in anxiety and depression, nurses must be equipped to deliver, or refer patients to, specific, evidence-based therapies like CBT or CBSM. This shifts the focus from asking *if* psychological support is helpful to defining *which* specific interventions are most effective.^[15]^

### 3.2. The role and nuances of technology-mediated interventions

The literature highlights the growing importance of delivering interventions via technology. A systematic review by Wan SW et al (2022) concluded that “Web-based interventions are effective in reducing depression, cancer-specific distress, and improving quality of life,” supporting the feasibility of digital platforms.^[12]^ However, a large European RCT by Maguire R et al (2021) introduces a critical nuance, finding that a remote symptom-monitoring system, while improving patient self-efficacy, showed “no significant differences in quality of life, anxiety, or depression.”^[14]^

This offers a mature insight into the role of technology: the technology itself is not the solution. Passive monitoring is insufficient to alter psychological outcomes. The intervention must contain an active therapeutic element. This reveals an important distinction for practice: nurses should prioritize adopting technologies that facilitate active therapeutic engagement (such as the successful blended CBT model used by Custers et al) rather than those that simply monitor symptoms.

### 3.3. The critical distinction between general psychoeducation and targeted therapy

The evidence reveals a critical distinction between the effectiveness of different types of psychological support. A major systematic review (Zhang X et al, 2023) found that while general psychoeducational interventions “significantly improved quality of life (QoL),” their effects on anxiety and depression “were not significant.”^[10]^ When contrasted with the positive results from the CBT/CBSM trials, this finding points to a vital conclusion: while broad educational support is beneficial for a patient’s overall well-being, more specific therapeutic techniques are necessary to treat clinical symptoms of anxiety and depression.

This distinction strongly suggests that a “stepped-care” model, as outlined in recent reviews and guidelines on managing cancer-related psychological distress,^[16,17]^ is the most logical approach for nursing. Under this model, all patients could receive psychoeducation for general well-being and improved QoL. However, patients identified with significant distress through routine screening would be “stepped up” to receive targeted, evidence-based therapies like CBT, which are required to effectively manage their more severe symptoms. This approach aligns perfectly with screening guidelines from bodies like the NCCN.^[7]^

### 3.4. Implications for clinical nursing practice

The evidence synthesized in this review offers clear and actionable recommendations for nursing:

1. Implement a stratified intervention model: Nursing practice should adopt a 2-tier approach. All patients receive psychoeducation to enhance QoL, while those with higher distress receive targeted therapies like CBT.^[18]^
2. Develop core competencies in evidence-based therapies: Nursing departments must invest in training nurses in the core skills of therapies like CBT to empower them to deliver effective care directly or make informed referrals.^[19]^
3. Use technology for therapy, not just monitoring: When adopting technology, nurses should choose tools that deliver active therapy, such as platforms supporting CBT, to overcome geographical barriers and increase access to care.^[20]^

### 3.5. Strengths and limitations

This review’s key strength is its strict focus on recent, high-level evidence, ensuring robust and clinically relevant conclusions. It also provides a balanced perspective by integrating studies with both positive and negative findings.^[21]^ However, the review has limitations. It is a narrative synthesis, not a quantitative meta-analysis. The conclusions are based on 5 key studies, and a larger evidence base would improve generalizability. Furthermore, a potential limitation is the geographic scope of the evidence, as the studies were predominantly conducted in European and Asian populations.^[22]^

### 3.6. Directions for future research

Based on this review, we propose 4 directions for future research:

1. Directly compare technology models: Conduct RCTs to test platforms with active therapeutic content against those with only passive monitoring.^[23]^
2. Develop and validate stepped-care protocols: Design and test formal stepped-care models for CRC patients, defining screening cutoffs for escalating care.^[24]^
3. Conduct health economic evaluations: Future studies must include cost-effectiveness analyses to demonstrate the financial value of investing in these nursing models.^[25]^
4. Perform cross-cultural adaptation studies: Adapt and test these effective interventions in diverse cultural settings to ensure global relevance.^[26,27]^

## 4. Conclusion

In conclusion, this review confirms that addressing psychological distress is an indispensable component of comprehensive CRC care. The synthesized evidence reveals a clear path forward, moving beyond passive problem Acknowledgments to the active implementation of a systematic psychosocial support pathway where nursing plays a central role.

The research shows that by using risk-stratified screening, it is possible to identify vulnerable patients and guide them toward the right level of care. Specifically, it highlights the distinction between providing broad psychoeducational support to improve quality of life for all patients, and delivering targeted, evidence-based therapies like CBT, which are required to effectively treat clinical distress. Investing in specialized training will empower nurses to become linchpins in this system. They can expertly manage this tiered approach and leverage technology – not just for monitoring, but as a scalable tool for delivering active, effective therapeutic interventions.

Therefore, integrating this structured and evidence-based approach to psychological care is not merely an optional enhancement to oncology services. It is a fundamental requirement for achieving truly holistic, patient-centered care and improving the long-term quality of life for all individuals navigating the challenges of CRC.

## Author contributions

**Investigation:** Ping Ding.

**Methodology:** Ping Ding.

**Project administration:** Ping Ding.

**Validation:** Qingbo Zhou.

**Writing – original draft:** Jiaying Hu.

**Writing – review & editing:** Jiaying Hu.
